# Surface interactions and radical generation in TCD decomposition: a DFT approach

**DOI:** 10.1007/s00894-025-06545-y

**Published:** 2025-10-27

**Authors:** Samantha E. Knoth, Daniel Tunega, Adelia J. A. Aquino

**Affiliations:** 1 Department of Mechanical Engineering, Texas Tech University, Lubbock, TX 79409 USA; 2 Institute for Soil Research, Department of Ecosystem Management, Climate and Biodiversity, BOKU University Vienna, Peter-Jordan-Strasse 82, 1190 Vienna, Austria

**Keywords:** TCD, H-abstraction, Absorption reaction, DFT, Defected γ-Al_2_O_3_ surface

## Abstract

**Context:**

Exo-tetrahydrodicyclopentadiene (exo-TCD) is a key component of Jet Propellant-10 (JP-10), a high-density hydrocarbon fuel extensively used in aerospace applications. The addition of aluminum particles enhances fuel performance and reactivity, making the understanding of initial decomposition pathways crucial. This study used density functional theory (DFT) calculations to investigate the initial hydrogen abstraction reactions in the decomposition of exo-TCD, with emphasis on radical formation processes. A significant aspect of this work is the role of the γ-Al_2_O_3_ surface in facilitating these reaction pathways, especially considering surface defects modeled by removing hydrogen from active hydroxyl groups. Five known active hydroxyl sites on γ-Al_2_O_3_ (A_Ia, A_Ib, A_IIA, B_IIb, and B_III) were used to construct complexes with exo-TCD. The formed complexes are primarily van der Waals interactions, with energies ranging from −11 to −20 kcal/mol and no substantial energy differences between configurations. The results indicate that hydrogen abstraction from the R4 site of exo-TCD is the most energetically favorable, owing to the molecular structure. Surface defects can boost reactivity by facilitating hydrogen abstraction, as seen in spontaneous H transfer to the active A_Ib site and low energetic barrier to the transition state of the H-abstraction of the B_IIb site. These findings improve the understanding of TCD decomposition and the catalytic role of γ-Al_2_O_3_, aiding the development of better propulsion fuels and energetic materials.

**Methods:**

The calculations used the Perdew–Burke–Ernzerhof PBE exchange–correlation functional with split-valence polarization (SVP) and triple-zeta valence polarization (TZVP) basis sets, combined with the resolution of identity (RI) method to accelerate four-center electron repulsion integrals. The PBE results were benchmarked with the hybrid meta-GGA functional M06-2X. Dispersion correction D3 was applied throughout. All computations were performed using the Turbomole program.

## Introduction

High-density hydrocarbon fuels are increasingly valued in the aerospace industry due to their ability to enhance payload capacity, flight range, and aircraft speed owing to their high energy density [1, 2]. Jet Propellant-10 (commonly referred to as JP-10) is a synthetic liquid aircraft and missile fuel produced from the hydrogenation of dicyclopentadiene [3] and primarily consists of *exo*-tricyclodecane (TCD) hydrocarbon also known as tetrahydrodicyclopentadiene, which makes up 96.5 wt%. Its minor components include endo-tetrahydrodicyclopentadiene (2.5%) and adamantane (1.0%), totaling 3.5%. Beyond its high mass density of 0.935 g·mL^−1^ at 20 °C, JP-10 also has a high volumetric energy density of 39.6 MJ·L^−1^ and a low freezing point of − 79 °C [4–7], which are desirable attributes for an aviation fuel. It is important to point out that its strained cyclic structure enhances its ability to store energy efficiently and provides exceptional thermal stability, rendering it suitable for missile propulsion [3, 8, 9].

Zehe and Jaffe [10] conducted high-level ab initio calculations to determine the heats of formation of the exo and endo isomers of gas-phase TCD. They employed the Gaussian Gx and Gx(MPx) composite methods, as well as the CBS-QB3 method. Additionally, the authors investigated several isodesmic and homodesmotic reaction schemes for the formation of TCD and its well-characterized isomer, adamantane. By combining the computed gas-phase heats of formation with experimental data on heats of vaporization, they proposed values for the standard enthalpy of formation at 298 K in the liquid state: −30.1 kcal/mol for the exo isomer and −27.4 kcal/mol for the endo isomer. The abstraction of a hydrogen atom from exo-TCD by hydroxyl radical in a set of twenty reactions was investigated through DFT using the hybrid functional M06-2X with MG3S basis set, by Wu et al. [11], aiming to predict the reaction kinetics. Their theoretical calculations of the rate constants for this reaction over the temperature range of 200–2000 K showed good agreement with experimental data. The hydrogen abstraction process and the subsequent formation of TCD radicals were investigated at a high ab initio level of theory using several DFT functionals and G3MP2B3 and CBS-QB3 composite computational chemistry methods. To reduce calculation errors, a series of five isodesmic reactions involving reference molecules with similar ring strain were employed. The enthalpy of formation for TCD was determined to be −19.5 kcal/mol, which is several kcal/mol lower than the commonly cited values in the literature [12]. A detailed experimental and theoretical study by Brotton et al. [13] on the combustion of exo-tetrahydrodicyclopentadiene doped with titanium–aluminum–boron (Ti–Al–B) reactive metal nanopowders concluded that the addition of these nanopowders to TCD not only produces a fuel with higher energy density but also is expected to result in shorter ignition delays compared to pure JP-10. This is attributed to the formation of highly reactive BO, AlO, and BO_2_ radicals during the initial stages of oxidation.

To improve JP-10 combustion properties, fuel additives, such as aluminum nanoparticles (nAl), were added to the fuel [14–17]. Fuel blends of JP-10 and nAl are currently being tested experimentally. In 2023, Biswas et al. [14] published results from experimentally testing JP-10 blended with nAl under pyrolysis conditions and uncovered an interesting finding: that some of the decomposition products captured and characterized contained oxygen. The authors concluded that oxygen present in the decomposition products likely originates from the γ-Al_2_O_3_ surface which envelops the core of nAl. The alumina shell was the only identifiable source of oxygen in the fuel mixture as no externally supplied oxygen-containing source was present [14]. This discovery requires further investigation to determine the pathways which facilitate deoxygenation of γ-Al_2_O_3_. This phenomenon of depleting oxygen from the passivation shell of nAl inherently makes the nAl more reactive, which will result in enhanced combustion in the engine.

**Scheme 1:**
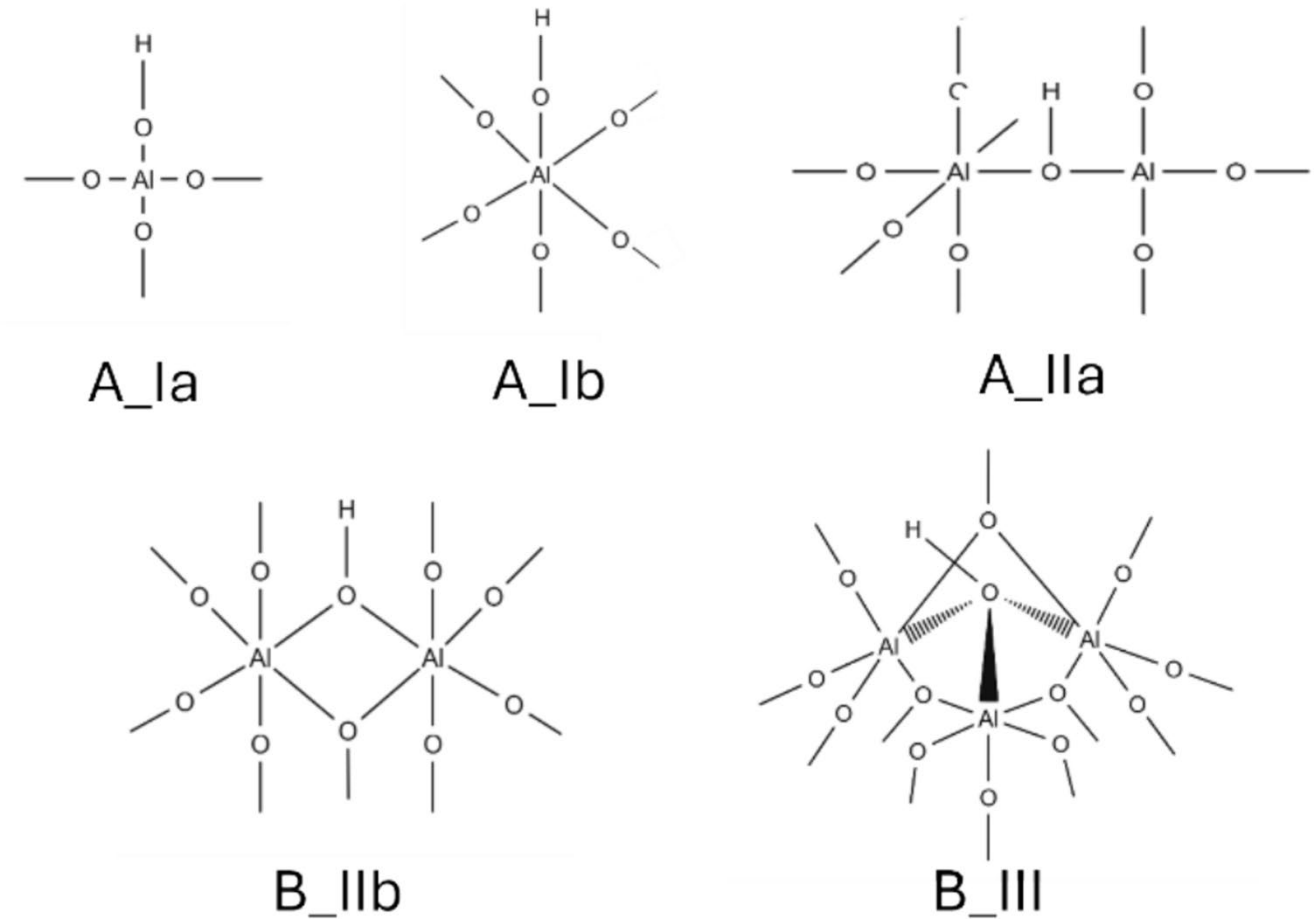
Five hydroxyl active sites of γ-Al_2_O_3_ surfaces

The γ-Al_2_O_3_ passivation shell, which serves as a protective barrier between the core of nAl and the surrounding environment—such as air—contains numerous active sites distributed across its surface. These active sites play a crucial role in surface reactions, including oxidation processes. It has been thoroughly established by Peri [18] and Knozinger and Ratnasamy [19] that the surface of γ-Al_2_O_3_ predominantly features five distinct types of hydroxyl-terminated active sites. These sites can be classified based on the bonding configuration of the hydroxyl group to the aluminum atoms. Specifically, there are terminal sites, where the hydroxyl group is bonded to a single aluminum atom in either tetrahedral or octahedral Al coordination, known as sites A_Ia and A_Ib respectively. There are also bridging sites, where the hydroxyl group is connected to two aluminum atoms (sites A_IIa and B_IIb). Additionally, edge sites involve the hydroxyl group being bonded to three aluminum atoms, classified as site B_III. These various surface sites are schematically represented in Scheme 1 and are critical in influencing the surface chemistry and reactivity of γ-Al_2_O_3_, particularly in catalytic and oxidation reactions.

This study is centered on employing density functional theory (DFT) calculations to investigate the initial pathways (H-abstraction) involved in the decomposition of exo-TCD, with a particular emphasis on the formation of radicals during the process. A key aspect of this work is to elucidate the role of the γ-Al_2_O_3_ surface in facilitating these reaction pathways. The computational results reveal detailed mechanisms by which the surface hydroxyl (OH) groups of γ-Al_2_O_3_ active sites participate in the cleavage of hydrogen atoms from the exo-TCD molecule, thereby initiating the decomposition process. Specifically, the interactions between the surface hydroxyl groups and the exo-TCD molecule enable H atom transfer reactions that are crucial for radical formation and subsequent reaction steps. Throughout the remainder of this paper, exo-TCD will be consistently referred to as TCD for simplicity. This research aims to deepen the understanding of how the γ-Al_2_O_3_ surface influences the decomposition pathways of TCD, which has significant implications for catalytic applications and combustion processes involving aluminum-based materials.

## Computational methodology

**Scheme 2:**
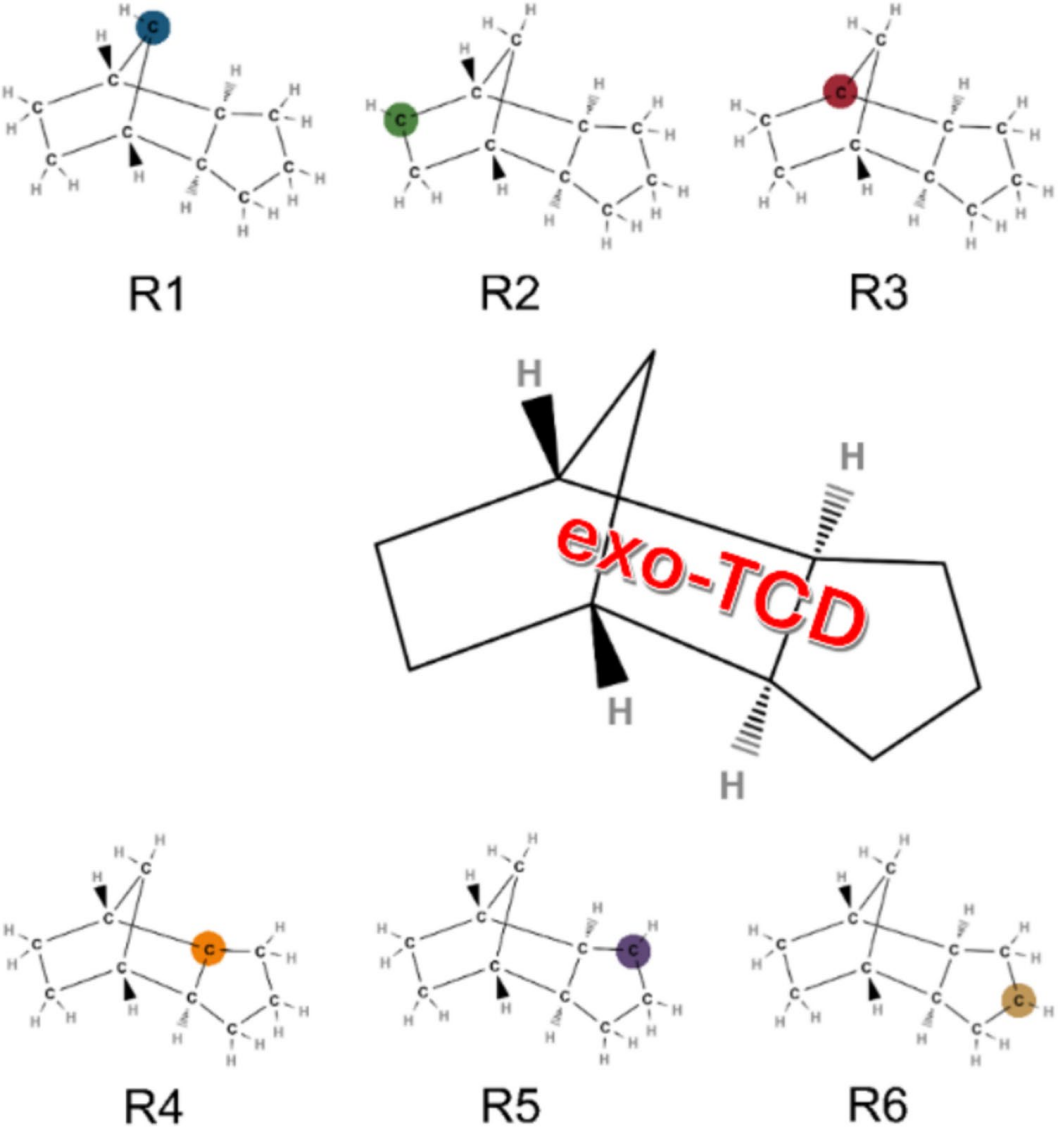
Molecular structures of neutral TCD and its radicals R1–R6

The molecular systems studied in this work were neutral TCD molecule and its radicals and molecular clusters of γ-Al_2_O_3_ surfaces representing OH active sites. Among the possible TCD conformers (*exo*, *endo*, boat, chair), we selected the most stable *exo*-TCD chair conformation, as determined in previous theoretical studies [10, 12]. The TCD radicals are formed by homolytic C–H bond breaking [11, 20]. Accounting for molecular symmetry, there are six unique radical forms of TCD identified as R1–R6. Scheme 2 shows the TCD structure in the center surrounded by the six TCD radicals, the radical carbon centers color coded.

Five γ-Al_2_O_3_ clusters (representing five different OH sites, Scheme 1) were the same as used in previous work by Padhye et al. [21]. These cluster models were derived from slabs created using experimental bulk structure of the (111) γ-Al_2_O_3_ surface, as determined by the single-crystal X-ray diffraction from Smrčok et al. [22]. To assure the clusters maintained an overall neutral charge, hydrogen atoms were added at the edges and to the dangling Al–O bonds, as H^+^ if linked to the oxygen or H^−^ if linked to the aluminum. Additionally, aluminum atoms from the γ-Al_2_O_3_ surface were constrained (with their coordinate positions fixed) during the calculations to preserve the crystal structure of γ-Al_2_O_3_ as much as possible, while all other atoms were allowed to relax. These clusters contain about 100–120 atoms in total and will be labeled as A_Ia, A_Ib, A_IIa, B_IIb, and B_III, according to sites shown in Scheme 1. More details on the cluster constructions can be found in the referred work [21]. γ-Al_2_O_3_ is known as a highly disordered structure, with defect sites at surface of its particles [23, 24]. These defected sites can serve as reaction centers that can activate the formation of the radicals of TCD molecule. Therefore, we created defected sites by removing H atom from the OH center and calculated interactions of exo-TCD with these defected γ-Al_2_O_3_ clusters. Note that those defected sites were not considered charged as in the experimental work on the TCD decomposition facilitated by Al_2_O_3_ supposed that no ionic species were involved in these reactions [14].

The initial calculations included geometry optimization of all isolated species. Then, the adsorption complexes were created by merging the optimized geometries of γ-Al_2_O_3_ and TCD species, aligning the TCD main rings nearly parallel with the surface of the γ-Al_2_O_3_ clusters at a quasi-perpendicular distance of approximately 2.5 Å using CrystalMaker software [25]. This initial configuration was estimated as the most stable van der Waals adsorption complex as the maximum contacts of CH units (H in *exo* positions) with the cluster are formed. No imaginary frequency was found for any of the systems investigated. Adsorption energies, Δ*E*_ads_, were calculated as a difference in the total energy of the complex and the sum of the total energies of the isolated species. Considering the weak interactions, basis set superposition error (BSSE) [26] was computed for the set of optimized geometries of the complexes representing adsorbed TCD molecules on all five non-defected clusters to assess the influence of the basis set on the adsorption energies. Further, atomic charges were calculated using the natural population analysis (NPA) [27] method to analyze charge transfer between TCD species and γ-Al_2_O_3_ clusters. Neutral species (exo-TCD molecule and all five non-defected clusters) were considered singlets, while R1–R6 radicals and defected clusters were considered doublets.

Calculations were performed at the DFT level of theory using the Perdew–Burke–Ernzerhof (PBE) [28, 29] exchange–correlation functional and two basis sets, split-valence polarization (SVP) [30] and triple-zeta valence polarization (TZVP) [31], including the resolution of identity (RI) method [32] to speed up the calculations of four-center electron repulsion integrals. The SVP basis set was used in the initial phase to accelerate geometry optimization. After reaching the geometry convergence with the SVP basis set, the optimization was extended using the TZVP basis set. Since the dominant interactions in the studied models are of nonbonding dispersion nature, the PBE results were validated by employing the hybrid meta-GGA functional of M06-2X type, which is known to better describe nonbonding dispersion interactions than standard GGA functionals [33]. Dispersion correction D3 by Grimme [34] was applied to all calculations. Note that in calculations with the M06-2X functional, D3 dispersion corrections were also involved but with a different parametrization [35]. The computations were performed using the Turbomole program suite [36, 37].

Since H-abstraction is a highly dynamic process, we also performed transition state (TS) calculations to estimate its kinetics. For this purpose, the TS optimization was carried out using the Trust Radius Image Minimization (TRIM) method [38], as implemented in the Turbomole package.

## Results and discussion

### Isolated species

Ground state geometry optimization was carried out for all isolated systems investigated, including neutral TCD and all its radicals R1–R6, and γ-Al_2_O_3_ clusters in the form of neutral clusters (complete OH surface) and corresponding models with defected OH sites (removed H atom).

The initial step involved calculating the C–H bond dissociation energies (BDE) for each TCD radical bond as well as the formation energy of defected γ-Al_2_O_3_ clusters by removing H from the OH site. The final calculations were performed using PBE-D3 and M06-2X-D3 functionals and the TZVP basis set. The BDE results for the TCD molecule are collected in Table 1 alongside data from the previous studies for reference [11, 12]. The BDE values showed a small difference between PBE-D3/TZVP and M06-2X/TZVP levels, with M06-2X-D3 values about 2–3 kcal/mol higher than those for PBE-D3. Furthermore, a comparison with the results collected from reference [12] revealed only a small difference (about 2–3 kcal/mol) between PBE-D3 results and the results achieved at the B3LYP/6–31 g(d,p) level or even more time-consuming high-level ab initio and DFT-based composite CBS-QB3/G3MP2B3 methods (for details on these methods, see references [39–41]). The trend in the BDE in our study is the same as observed in the previous studies, showing the largest BDE for the site R1 and the lowest BDE for the site R5. However, the BDEs are within a narrow range of energies (approximately 5 kcal/mol), indicating that the first step of TCD decomposition (H release) has a similar probability for each H position. Indeed, it will be shown later that not all sites are in direct contact with the γ-Al_2_O_3_ surface and, therefore, are not equally influenced by defect sites.

**Table 1:** PBE-D3/TZVP and M06-2X/TZVP calculated BDE (in kcal/mol), NPA atomic charge (*q*(C^*^)), and spin density localization (ρ(C^*^)) of the C^*^ radical site of six possible radicals of TCD molecule and their comparison with the previous works (D3 is not included in PBE and M06-2X label)

Rx	PBE	M06-2X	B3LYP/6–31 g(d,p)^a^	CBS-QB3/G3MP2B3^a^	M06-2X/MG3S^b^	*q*(C^*^), |*e*|		ρ(C^*^), |*e*/bohr^3^|	
						PBE	M06-2X	PBE	M06-2X
R1	106.9	108.6	104.0	104.1	104.3	−0.09	−0.07	0.82	0.86
R2	101.9	104.5	98.8	98.7	99.0	−0.11	−0.08	0.81	0.86
R3	109.4	111.8	106.9	107.2	107.4	0.15	0.17	0.75	0.80
R4	102.0	105.1	98.9	100.1	100.1	0.15	0.18	0.76	0.81
R5	100.6	103.1	97.6	98.0	97.9	−0.09	−0.07	0.81	0.86
R6	101.1	104.0	98.1	98.5	98.4	−0.10	−0.08	0.83	0.87

^a^Ref. [12]; ^b^Ref. [11]

Furthermore, Table 1 presents results for the calculated atomic charges and the spin density localization associated with the C^*^ radical site of the R1–6 species, obtained through the natural population analysis (NPA). In the neutral TCD molecule, the NPA charges on the C atoms typically range from approximately −0.2 to −0.4 |*e*|, indicating a moderate level of electron density localized on these carbons. Conversely, the H atoms have charges around 0.2 |*e*|, consistent with their typical electronic environment. At the radical C^*^ site, the atomic charge decreases significantly, oscillating around 0.0 |*e*|, which reflects a redistribution of electron density associated with radical formation. The spin density analysis reveals that the unpaired electron is predominantly localized on the radical site, as evidenced by the high spin density value (Table 1). The atomic charges of the remaining C and H atoms in the R1–6 radicals are quite similar to those of the neutral TCD molecule.

In the next step, we calculated BDE for all five active OH sites on the γ-Al_2_O_3_ clusters (Scheme 1). These defected sites are expected to play an active role in the abstraction of H^*^ from the TCD molecule. The PBE-D3 and M06-2X-D3 results are summarized in Table 2. There is a difference between both methods; M06-2X-D3 provides larger BDE values than PBE-D3. Differences in BDE among all five sites are likely due to the binding of the OH group to different numbers and types of Al atoms (Scheme 2). Furthermore, the defected O sites are not isolated and can be stabilized by H-bonds from the neighboring OH sites of the clusters, which may also influence the BDEs and can also explain the difference between PBE-D3 and M06-2X-D3 results. The highest BDEs were observed for the terminal OH group of site A_Ia (OH bound to a single Al_IV_ atom) and bridged OH of site B_IIb (OH bound to two Al_VI_ atoms). The most significant difference is seen for the B_III site, which is triply coordinated to Al atoms; here, the BDE is approximately 20–30 kcal/mol lower than for the other four sites. This suggests that the releasing of an H atom from the B_III site requires the least energy, making it the most accessible and probable site for H-abstraction among all five OH sites.

**Table 2:** BDE (kcal/mol) for active OH sites of γ-Al_2_O_3_ clusters calculated at PBE-D3 and M06-2X-D3 levels of theory (both TZVP basis set)

Site	PBE-D3	M06-2X
A_Ia	127.9	132.1
A_Ib	120.0	122.6
A_IIa	111.6	122.9
B_IIb	108.4	132.5
B_III	72.0	100.5

### Adsorption complexes of TCD molecules on non-defected γ-Al_2_O_3_ clusters

Following this, the focus of the investigation now was on determining the adsorption energies for the neutral TCD molecule on all five non-defected γ-Al_2_O_3_ clusters to gain insight into their interaction strength. The initial placement of the TCD molecule was carefully chosen based on the area of maximum contact between the molecule and cluster surface(s). Specifically, positions where the two main C-rings (Scheme 2) were oriented quasi-parallel to the surface of each cluster were selected. These initial configurations aimed to mimic the most probable adsorption sites by maximizing surface interaction.

**Fig. 1:**
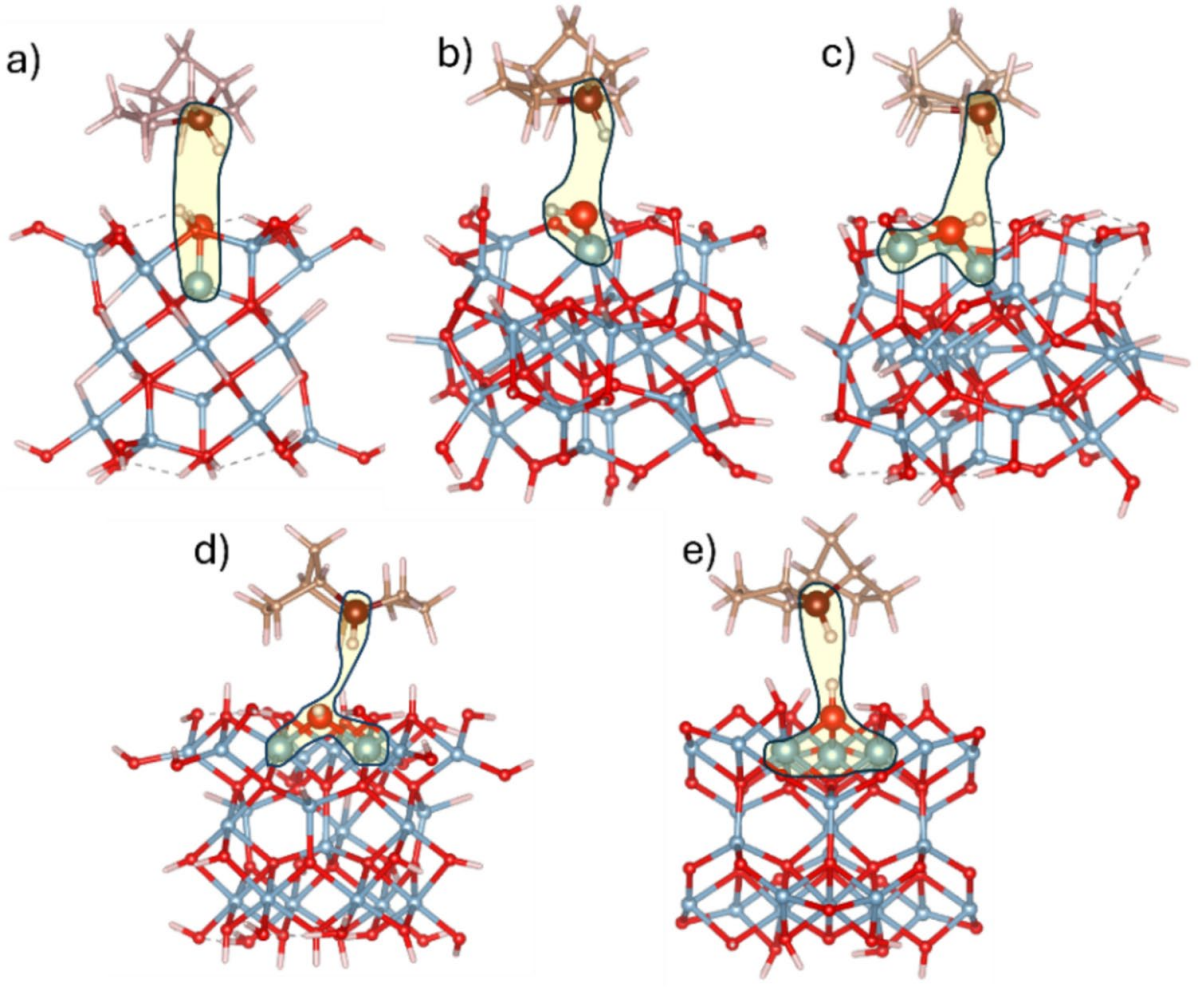
Structures of optimized geometries of TCD molecule adsorbed on γ-Al_2_O_3_ clusters representing five Al_x_-O-H centers (Scheme 1) (**a**) A_Ia (*x* = 1), **b** A_Ib (*x* = 1), **c** A_IIa (*x* = 2), **d** B_IIb (*x* = 2), and (**e**) B_III (*x* = 3). Letter *x* indicates the number of Al atoms bound to OH group. The Al_x_-O-H centers and the CH group of the TCD molecule closest to the OH site are shown as large balls and highlighted with a yellow area. Color scheme: Al atoms, blue; O atoms, red; C atoms, brown; and H atoms, white

Subsequent geometry optimization was performed under constraints that fixed Al atoms in the γ-Al_2_O_3_ clusters, as described in the Computational Details section. During optimization, the TCD molecule experienced slight shifts and rotations but maintained a predominantly parallel orientation relative to the surface. All five final PBE-D3/TZVP optimized geometries are collected in Fig. 1. Further M06-2X-D3/TZVP optimization resulted in minimal changes in the final optimized geometries. The resulting adsorption complexes are characterized as weak van der Waals interactions, reflecting a physisorption nature. This is consistent with the fact that the TCD molecule is essentially nonpolar, whereas the surface hydroxyl groups of the γ-Al_2_O_3_ clusters are polar. As a result, the surface OH groups induce a slight polarization in the adsorbed TCD molecule.

The average distance between the plane formed by the carbon atoms of TCD’s two main rings and the plane of the topmost Al layer of the γ-Al_2_O_3_ clusters was found to be approximately 3.8–4.3 Å.

The calculated adsorption energies of the neutral TCD molecule on non-defected clusters with three used methods are presented in Table 3. Recognizing that weakly bound molecular complexes are susceptible to basis set superposition error (BSSE), particularly when small basis sets such as SVP are employed, BSSE corrections were performed for these systems. These were calculated as single-point energy corrections on the optimized adsorption complexes and are also included in Table 3 for comparison. The results clearly show that BSSE is sizable for the SVP basis set, with values ranging from 5 to 8 kcal/mol. In contrast, the larger TZVP basis set significantly reduced BSSE to around 1–3 kcal/mol for both PBE-D3 and M06-2X-D3 methods. After applying BSSE corrections, the adsorption energies in Table 3 obtained with all methods became comparable. The corrected values for the TZVP basis set indicate consistently moderate adsorption energies across all five clusters, ranging from −9.1 to −12.8 kcal/mol (PBE-D3 level in Table 3). This consistency suggests a similar interaction strength of TCD with the non-defected γ-Al_2_O_3_ surfaces, regardless of the specific cluster or the basis set used. The M06-2X-D3 method provided very similar results for adsorption energies as the PBE-D3. Based on the calculated NPA charges (TZVP basis set), we determined the charge transfer (CT) from the TCD molecule to the neutral γ-Al_2_O_3_ clusters (Table 3, last two columns). The results clearly indicate that the charge transfer is minimal, as evidenced by the small values of CT. Again, both PBE-D3 and M06-2X-D3 yielded similar results. Therefore, the adsorption energies and the charge transfer analyses indicated that PBE-D3 also provides acceptable results for the studied complexes, where dispersion interactions play a dominant role.

**Table 3:** Calculated adsorption energies, Δ*E*_ads_ (kcal/mol), for TCD molecule adsorbed on non-defected (neutral) γ-Al_2_O_3_ corrected with BSSE (BSSE is in ()). The last two columns contain calculated charge transfer from the TCD molecule to γ-Al_2_O_3_ clusters with TZVP basis set

Site	Δ*E*_ads_ (BSSE)			CT |*e*|	
	PBE-D3/SVP	PBE-D3/TZVP	M06-2X-D3/TZVP	PBE-D3	M06-2X-D3
A-Ia	−9.27 (8.02)	−10.28 (1.78)	−9.47 (1.51)	0.013	0.013
A-Ib	−8.16 (5.23)	−9.12 (1.67)	−8.57 (1.63)	0.020	0.019
A-IIa	−9.27 (6.45)	−9.95 (1.77)	−7.56 (2.17)	0.022	0.034
B-IIb	−11.97 (7.01)	−12.80 (2.55)	−12.24 (2.60)	0.025	0.027
B-III	−10.87 (6.50)	−11.73 (1.99)	−13.09 (0.73)	0.033	0.028

In the following sections, only the results obtained with the TZVP basis set will be discussed.

### Adsorption complexes of TCD molecule on O sites of defected γ-Al_2_O_3_ clusters

**Fig. 2:**
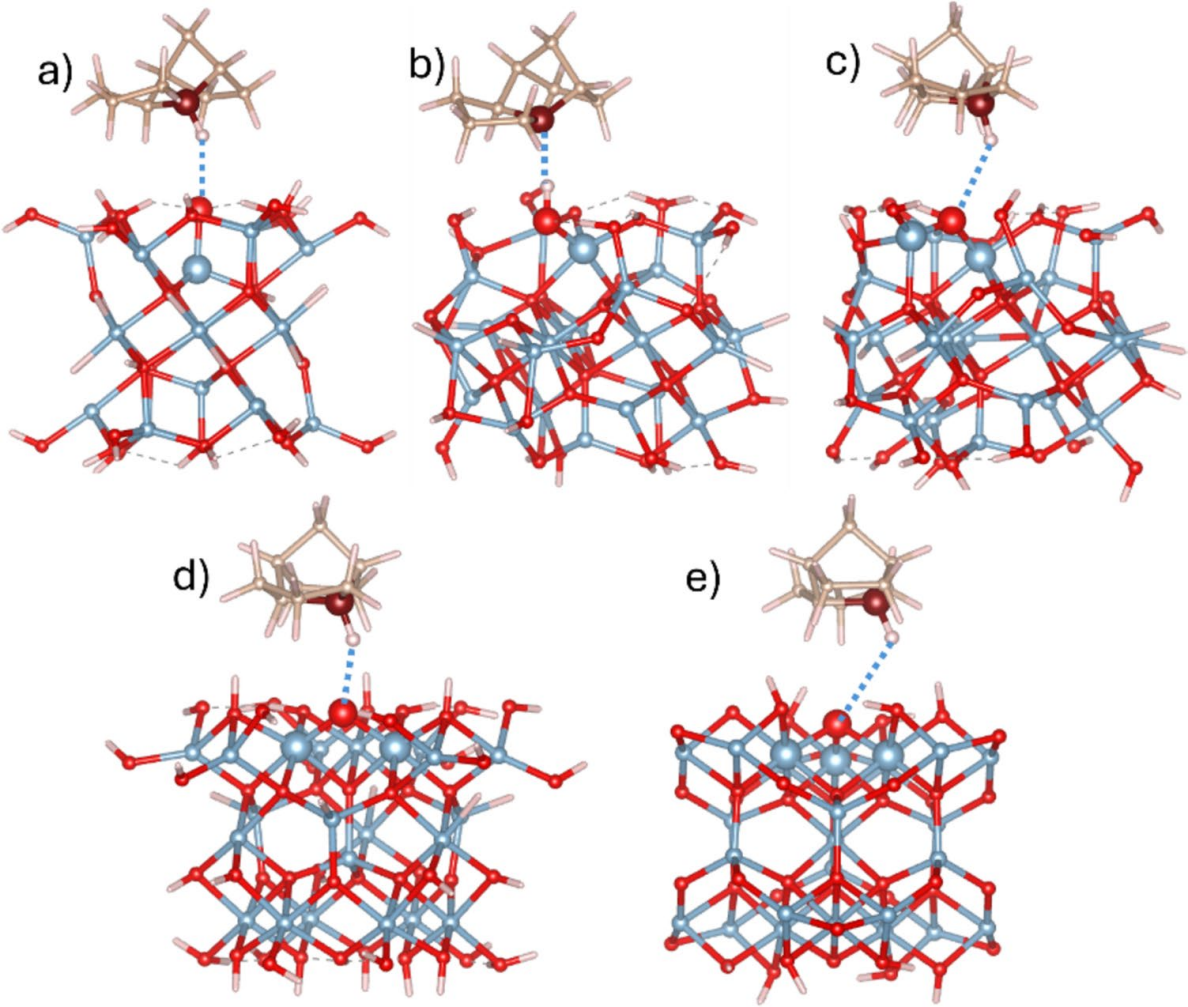
Structures of optimized geometries of TCD molecule adsorbed at defected γ-Al_2_O_3_ clusters representing five Al_x_-O centers (**a**) A_Ia (*x* = 1), **b** A_Ib (*x* = 1), **c** A_IIa (*x* = 2), **d** B_IIb (*x* = 2), and (**e**) B_III (*x *= 3). The Al_x_-O centers and the R4 CH site are represented as large balls. *d*(C–H···O_cl_) distance (Table 4) is shown as blue dotted line. Color scheme: Al atoms, blue; O atoms, red; C atoms, brown; and H atoms, white

The defected sites of γ-Al_2_O_3_ were created by removing a hydrogen atom from the active OH center (see Scheme 1). The positions of the TCD molecule were the same as in the optimized adsorption complexes of the neutral TCD molecule and non-defective γ-Al_2_O_3_ clusters, as discussed in the previous section. Geometry optimization of these configurations yielded surface complexes in which the CH group of the R4 site (Scheme 2) was the closest to the oxygen vacancy defect (O_cl_) of the γ-Al_2_O_3_ clusters (Fig. 2). The distances obtained at the PBE-D3/TZVP level are collected in Table 4 having a range narrowly from 2.34 to 2.64 Å, with the shortest distance observed for the B_IIb site (a bridged O site bound to two sixfold coordinated Al atoms—Al_VI_), and the longest distance was found for the A_IIa site (bridged O site bound to one sixfold and one fourfold coordinated Al atoms). The distances obtained after the M06-2X-D3/TZVP optimization changed minimally compared to the PBE-D3/TZVP results (Table 4). The PBE-D3 and M06-2X-D3 calculated adsorption energies of the TCD molecule on all five defected γ-Al_2_O_3_ clusters are also provided in Table 4. The consistency between PBE-D3 and M06-2X-D3 results is also observed for these models.

**Table 4:** PBE-D3/TZVP and M06-2X-D3/TZVP calculated adsorption energies (in kcal/mol), and measured *d*(C–H···O_cl_) distance for the optimized clusters of the TCD molecule adsorbed the defected γ-Al_2_O_3_ clusters. The last two columns show the calculated charge transfer from the TCD molecule to the defected γ-Al_2_O_3_ clusters

Site	Δ*E*_ads_, kcal/mol		*d*(C–H···O_cl_), Å		CT |*e*|	
	PBE-D3	M06-2X-D3	PBE-D3	M06-2X-D3	PBE-D3	M06-2X-D3
A_Ia	−11.99	−12.20	2.58	2.59	0.042	0.010
A_Ib^a^	−30.83	−28.66	-	-	0.531	0.529
A_IIa	−11.69	−11.28	2.69	2.68	0.020	0.021
B_IIb	−15.94	−14.96	2.36	2.27	0.064	0.044
B_III	−15.44	−11.95	2.64	2.56	0.020	0.011

^a^Spontaneous H transfer from R4 CH site to the O site of the A_Ib cluster

Except for the site A_Ib (O site bound to one Al_VI_, see Scheme 1), the largest (in absolute value) adsorption energy is observed for the B_IIb site; however, the variation in adsorption energies across different sites is relatively small, with a difference of about 4.3 kcal/mol. The calculated adsorption energy for the A_Ib site referenced to the energy of the TCD molecule and defected cluster, as with the other four cases, appears significantly larger than the others. Yet, during the geometry optimization for this site (using both basis sets), a spontaneous hydrogen transfer from the R4 CH group to the O atom of the defected cluster was observed, resulting in the formation of the R4 radical from the TCD molecule and neutral, non-defected A_Ib cluster. This observation indicates that the O_cl_ site of the A_Ib cluster has the highest ability among all five sites to abstract a hydrogen atom from the R4 position. It is important to note that the adsorption energies presented in Table 4 are without BSSE corrections. We expected that the BSSE magnitude will be similar to those observed for the adsorption of neutral TCD species on the neutral clusters (Table 3). The difference in the adsorption energies of defected (Table 4) and corresponding non-defected γ-Al_2_O_3_ clusters (Table 3) is small (except the site A_Ib). However, it can be expected that the H-abstraction by defected O sites can be easier than by the OH group of the non-defected structures.

The calculated adsorption energies of the TCD molecule on defected clusters are comparable with values reported in the literature for the H-abstraction of TCD by OH radicals [11] and/or gaseous aluminum monoxide, AlO [13]. For example, the study [11] reported an adsorption energy of −18.3 kcal/mol for the R4 (level) site, which is comparable and aligns well with our values ranging from approximately −12 to −16 kcal/mol for most defected clusters (excluding A_Ib), as shown in Table 4. Similarly, the formation energy of −7.9 kcal/mol of the TCD complex (R4 site) with AlO monoxide (B3LYP-D3/def2-TZVP) [13] is not far from our results in Table 4.

The calculated CT between the TCD molecule and the defected γ-Al_2_O_3_ clusters (Table 4) is observed to be slightly higher than the TCD values obtained for the TCD adsorbed on the neutral γ-Al_2_O_3_ clusters (Table 3). This increase suggests that the presence of defects can enhance interaction or facilitate a greater degree of electronic exchange between the molecule and the surface. However, an important exception to this trend is the complex involving the adsorbed TCD on the A_Ib site. In this case, the CT value is notably larger, which can be attributed to a spontaneous H transfer from the R4 site to the defected cluster. This process not only increases the charge transfer but also indicates a more reactive and chemically active interaction at this specific site, emphasizing the significance of defect sites in modulating surface chemistry and molecular adsorption behavior.

### Adsorption complexes of R1–R6 TCD radicals on neutral γ-Al_2_O_3_ clusters

**Fig. 3:**
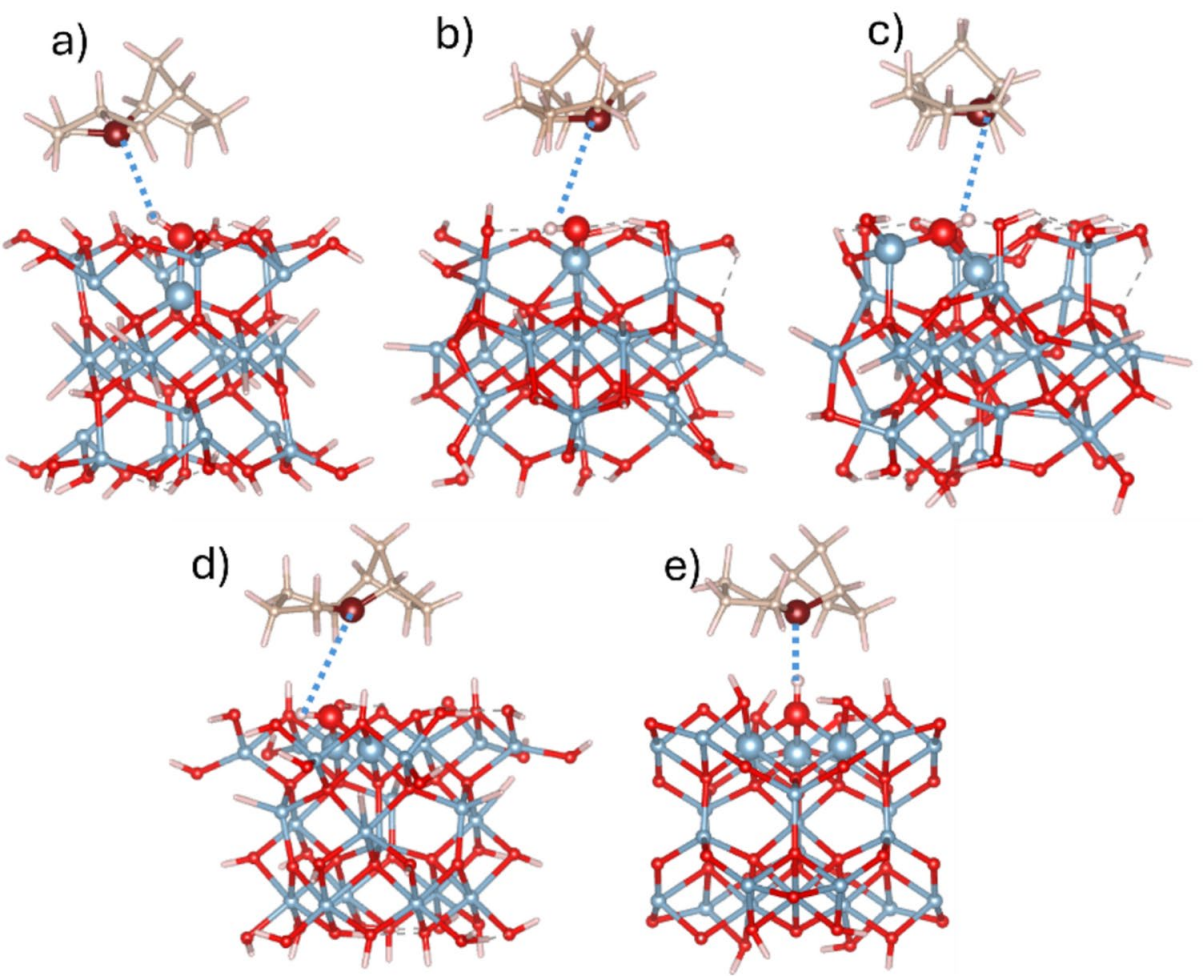
Structures of optimized geometries of R4 radical adsorbed at non-defected γ-Al_2_O_3_ clusters representing five Al_x_-O centers (**a**) A_Ia (*x* = 1), **b** A_Ib (*x* = 1), **c** A_IIa (*x* = 2), **d** B_IIb (*x* = 2), and (**e**) B_III (*x* = 3). The Al_x_-O centers and the R4 CH site are represented as large balls. *d*(C–H···O_cl_) distance (Table 4) is shown as a blue dotted line. Color scheme: Al atoms, blue; O atoms, red; C atoms, brown; and H atoms, white

Given the observation of a spontaneous hydrogen transfer at the R4 site of the TCD molecule, we also conducted calculations on four additional models where the R4 radical was adsorbed onto neutral, non-defected γ-Al_2_O_3_ clusters. The BPE-D3 and M06-2X-D3 results, including interatomic distances between C^*^ radical site of the R4 and active OH groups of five clusters, as well as the corresponding adsorption energies, are summarized in Table 5. The measured distances indicated that, for sites A_Ib, A_IIa, and B_IIb (bridged OH bound to two Al_VI_ atoms, Scheme 1), the C^*^···H–O_cl_ distance exceeds 3.2 Å, whereas for site A_Ia, the distance is approximately 2.7 Å. These relatively large distances are due to the flexibility of these OH groups, which can form hydrogen bonds with neighboring OH groups on the clusters, as illustrated in Fig. 3. On the other hand, the B_III site features an OH group bound to three Al_VI_ atoms, which is relatively rigid. This group is oriented perpendicularly to the cluster surface and does not participate in hydrogen bonding as the four other groups. Consequently, the R4 radical can approach the surface more closely, resulting in a much shorter C^*^···H–O_cl_ distance of about 2.05 Å (see Table 5 and Fig. 3e).

**Table 5:** PBE-D3/TZVP and M06-2X-D3/TZVP calculated adsorption energies and measured *d*(C^*^···H–O_cl_) distances for radical R4 adsorbed on all non-defected γ-Al_2_O_3_ clusters. The last four columns contain CT from R4 radical to γ-Al_2_O_3_ clusters and spin density localized at the C^*^ radical site of the R4 radical (D3 is omitted from the functional label)

Site	Δ*E*_ads_, kcal/mol		*d*(C^*^···H–O_cl_), Å		CT, |*e*|		ρ(C^*^), |*e/*bohr^3^|	
	PBE	M06-2X	PBE	M06-2X	PBE	M06-2X	PBE	M06-2X
A_Ia	−14.09	−14.73	2.69	2.64	0.012	0.016	0.737	0.79
A_Ib	−14.34	−13.49	3.41	3.29	0.040	0.030	0.697	0.76
A_IIa	−11.34	−11.90	3.46	3.42	0.022	0.025	0.759	0.80
B_IIb	−13.95	−14.21	3.66	3.57	0.026	0.030	0.767	0.82
B_III	−19.50	−18.14	2.09	2.08	0.063	0.052	0.666	0.74

The calculated adsorption energies in Table 5 (without BSSE correction) are not significantly different from the adsorption energies of the TCD molecule on defected clusters (Table 4). The strongest adsorption was observed for the B_III site that corresponds to the observed shortest C^*^···H–O_cl_ distance. The calculated CT for the R4 radical adsorbed on the non-defected clusters (Table 5) is similar to that of the adsorbed TCD molecule on the defected γ-Al_2_O_3_ clusters (Table 4). The calculated spin density values are predominantly localized at the C* site, similar to what was observed for the isolated R1–R6 radicals (Table 1). The differences in adsorption energies, distances, charges, and charge densities between PBE-D3 and M06-2X-D3 functionals are small, confirming the consistency of our previous observations.

**Fig. 4:**
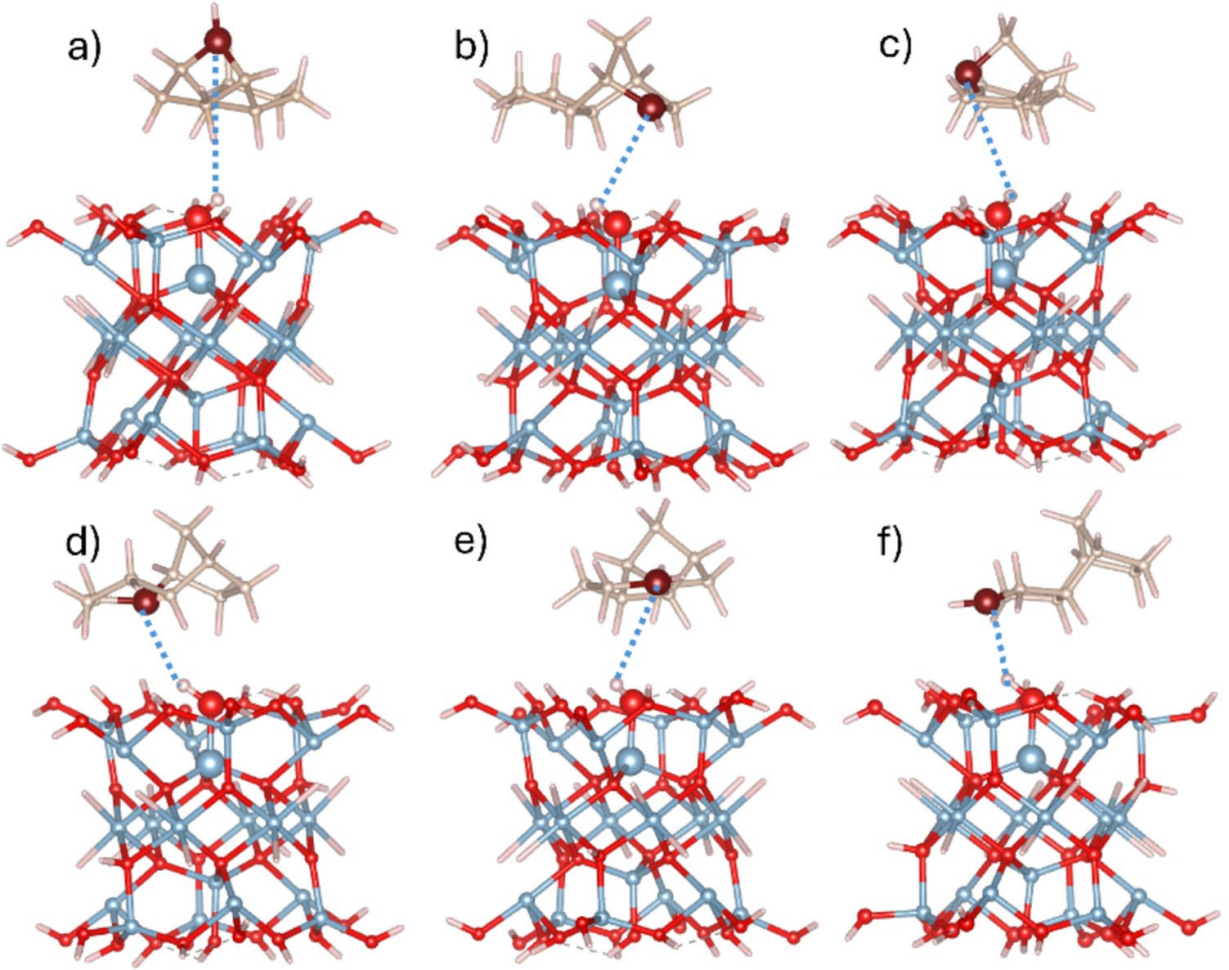
Structures of optimized geometries of R1–6 radical sites adsorbed at non-defected A_Ia γ-Al_2_O_3_ cluster (**a**–**f**) R1–R6. The Al_x_-O center and the Rx C^*^ sites are represented as large balls. *d*(C^*^···H–O_cl_) distance (Table 6) is shown as blue dotted line. Color scheme: Al atoms, blue; O atoms, red; C atoms, brown; and H atoms, white

Calculations were also performed on all six possible TCD radicals (Scheme 2) to examine differences among them, as the differences in BDE (Table 1) are small, indicating that the probability of each radical is similar. For this purpose, only one cluster representing the A_Ia site was selected. Table 6 presents the results of adsorption energies and distances achieved with the PBE-D3 and M06-2X-D3 functionals (TZVP basis set), and Fig. 4 presents the corresponding PBE-D3 optimized geometries. The energies are without the BSSE correction, as explained previously.

**Table 6:** PBE-D3/TZVP and M06-2X-D3/TZVP calculated adsorption energies (Δ*E*_ads_) and *d*(C^*^···H–O_cl_) distances for radicals R1–6 adsorbed on the A_Ia non-defected γ-Al_2_O_3_ cluster

C^*^ site	Δ*E*_ads_, kcal/mol		*d*(C^*^···H–O_cl_), Å	
	PBE-D3	M06-2X-D3	PBE-D3	M06-2X-D3
R1	−12.01	−10.86	4.63	4.63
R2	−12.45	−11.91	3.55	3.55
R3	−11.93	−10.75	3.89	3.93
R4	−14.09	−14.73	2.69	2.64
R5	−12.02	−11.00	3.29	3.30
R6	−12.83	−12.69	2.42	2.38

The distances between the C^*^ radical site of six TCD radicals and the active OH group of the γ-Al_2_O_3_ clusters do not represent the shortest path but rather reflect the positions of the radical site relative to the OH group. Although the adsorption energies of all R1–6 TCD radicals are very similar and comparable with the adsorption energies of other models, the differences in their distances are more pronounced. The strongest complex is formed by the R4 radical site. The largest variation in distances is observed for the R1 site, which is positioned at the top of the bridged ring of the TCD molecule (Scheme 1). Similarly, a large distance is also observed for the R3 site (Scheme 1). Moreover, this site also has the highest BDE (Table 1). This suggests that, due to steric effects, sites R1 and R3 cannot be affected by the presence of the γ-Al_2_O_3_ surface. PBE-D3 and M06-2X-D3 functionals provided similar results on adsorption energies and/or *d*(C^*^···H–O_cl_) distances.

Finally, the reaction energies, Δ*E*_r_, for the formation of the radical R4 from the adsorbed TCD molecule on the defected cluster surfaces were calculated, involving H-abstraction by the O_cl_ defect site within the adsorption complex of the defected clusters. The reaction can be written as TCD@def_γ-Al_2_O_3_ → R4@γ-Al_2_O_3_, where TCD@def_γ-Al_2_O_3_ (reactant) represents complexes of the TCD adsorbed on the defected clusters (Fig. 2), and R4@γ-Al_2_O_3_ (product) represents complexes of R4 radical with the neutral γ-Al_2_O_3_ clusters (Fig. 3). Then, Δ*E*_r_ is the energy difference between the R4@γ-Al_2_O_3_ complexes and the corresponding TCD@def_γ-Al_2_O_3_ complexes.

The reaction energies calculated using two functionals are listed in Table 7. Although the trend for reaction energies from A_Ia to B_III site is similar, relatively large differences are observed between the PBE-D3 and M06-2X-D3 results. This discrepancy may arise from small differences in the optimized geometries of the complexes obtained with PBE-D3 and M06-2X-D3, such as those related to hydrogen bonding.

**Table 7:** PBE-D3/TZVP and M06-2X-D3/TZVP calculated reaction energies as a difference of the energy of the complex of R4 and the neutral γ-Al_2_O_3_ cluster, and the energy of the adsorbed neutral TCD molecule at the defected γ-Al_2_O_3_ cluster

Site	Δ*E*_r_, kcal/mol	
	PBE-D3	M06-2X-D3
A_Ia	−21.78	−29.56
A_Ib	0.0	0.0
A_IIa	−9.24	−18.47
B_IIb	−11.84	−26.66
B_III	15.94	−1.61

Since the reaction involves only H transfer from the CH site to the O_cl_ defect, significant differences in thermal contributions to the calculated reaction energy are not expected, so the reaction’s thermodynamics could be estimated. According to the M06-2X-D3/TZVP reaction energies in Table 7, the formation of the R4 radical could be thermodynamically favorable for all sites except the site B_III. For this site, it is difficult to make a conclusion as the M06-2X-D3 energy is close to zero (although negative), while the PBE-D3 result is positive. Note that the difference for the A_Ib site is 0.0 as, during the optimization of the adsorbed TCD molecule at the defected A_Ib site, the spontaneous H transfer to the O_cl_ defect was observed and the R4 radical adsorbed on the non-defected cluster representing the A_Ib site was formed (discussed in Section “Adsorption complexes of TCD molecule on O sites of defected γ-Al2O3 clusters”).

### Transition states for reactions TCD@def_γ-Al_2_O_3_ → R4@γ-Al_2_O_3_

For a better understanding of the reaction dynamics of the H-abstraction from the R4 site of the TCD molecule to the oxygen sites (O_cl_) of the defected γ-Al_2_O_3_ clusters, we performed additional calculations to locate a transition state. Since transition state (TS) and frequency calculations are demanding and computationally intensive, the TS calculations were performed at the PBE-D3/SVP only. Linear interpolation between optimized geometries of reactant (TCD@def_γ-Al_2_O_3_) and product (R4@γ-Al_2_O_3_) was used to estimate a potential reaction pathway by generating 14 interpolated geometries. Single-point energy calculations were performed on these geometries, and the configuration with the highest energy (least negative) was selected as the initial estimate of the TS structure. Frequency calculation was then performed on this estimated TS configuration, yielding several negative frequencies. Of these, the highest in absolute value (approximately more than −1000 cm^−1^) was identified. Visualization of this vibrational mode demonstrated that it corresponds to the oscillation of the hydrogen atom between the carbon active site of the TCD molecule and the oxygen active site of the γ-Al_2_O_3_ clusters, accurately representing the transition vector. The TS optimization on the selected configuration was carried out using the Trust Radius Image Minimization (TRIM) method (Chem. Phys. Lett., 182(5), 503–510, (1991)), as implemented in Turbomole, based on the identified transition vector.

All TS calculations were performed for four sites (A_Ia, A_IIa, B_IIb, and B_III). For the site A_Ib, the TS calculation was not performed because spontaneous H transfer from the TCD molecule to the O-defected site was observed during geometry optimization, suggesting that the barrier for H transfer at this site is likely very low. This also indicates that the barriers for H-abstraction at other sites may be comparably low. For three sites (A_Ia, A_IIa, and B_III), the TS optimization failed; the negative frequencies disappeared, and the structures ultimately converged to the product complex (R4@Al_2_O_3_). For the site B_IIb, the optimization successfully identified a TS configuration, confirmed by a single negative frequency (−722.38 cm^−1^). The energy profile indicated that the reactant-to-TS barrier was very low, only 2.4 kcal/mol, supporting the hypothesis that the barriers for H-abstraction are generally small. This low barrier can also explain the failure of TS searches at the other three sites and aligns with the observed spontaneous H transfer during the geometry optimization of the TCD@A_Ib site. In the final TS structure, the TCD molecule was pulled closer to the B_IIb cluster, with a C···H distance of 1.28 Å and H···O distance of 1.41 Å. The TS results for the B_IIb site suggest that reactive oxygen sites on γ-Al_2_O_3_ surfaces significantly facilitate hydrogen abstraction from the TCD molecule.

## Conclusions

In this work, we conducted a comprehensive theoretical investigation into the adsorption behavior of the exo-TCD molecule, which is the main component of the JP-10 liquid rocket fuel, on cluster models that represent five different OH sites of the γ-Al_2_O_3_ surfaces. Our calculations were performed using the PBE-D3 and M06-2X-D3 density functional theory methods combined with the SVP and TZVP basis sets, allowing for accurate modeling of dispersion interactions critical for physisorption processes. The hybrid meta-GGA M06-2X-D3 functional was used for the validation of the PBE-D3 results. The results obtained from both functionals showed good agreement.

The primary aim of our work was to understand how surface defects influence adsorption characteristics, achieved by simulating defected surfaces through the removal of H atoms from the active OH groups, thus creating O active sites. Additionally, the dynamics of H-abstraction were examined through transition state calculations.

Multiple scenarios were explored to capture the diverse interactions: first, the adsorption of neutral TCD molecules on pristine, non-defected γ-Al_2_O_3_ clusters; second, the adsorption on defected clusters where H removal altered the surface chemistry; and third, the adsorption of six potential radical forms of TCD, representing various reactive intermediates, on the non-defected γ-Al_2_O_3_ surfaces. Our analysis revealed that the complexes formed have adsorption energies ranging from approximately −11 to −20 kcal/mol, indicating relatively weak but significant physisorption. The primary driving force behind the adsorption of TCD on γ-Al_2_O_3_ clusters appears to be dispersion interactions. This conclusion is supported by the observation of low values in the calculated charge transfer between TCD species and γ-Al_2_O_3_ clusters. Notably, the energy differences among these various types of complexes were modest, suggesting a degree of energetic equivalence in their stability.

A key finding of our study was that H-abstraction from the R4 site of the TCD molecule is energetically the most favorable pathway. This preference is largely attributed to the molecular structure of exo-TCD, which facilitates easier hydrogen transfer at this specific position. Furthermore, our calculations demonstrated that all types of oxygen defect sites could effectively promote H-abstraction from the TCD molecule. Particularly, the spontaneous proton transfer events observed during some simulations imply that certain defect sites, especially site A_Ib, exhibit heightened reactivity. The TS calculations were successful only for the B_IIb, which showed a low energy barrier of 2.4 kcal/mol between the reactant and transition state. For other sites, the TS optimizations finished in the product structure. The low energetic barrier at the B_IIb site and the spontaneous H transfer at the A_Ib site highlight the high reactivity of oxygen sites on defected γ-Al_2_O_3_ surfaces, which significantly facilitate hydrogen abstraction from the TCD molecule.

Our theoretical findings highlight the critical role of surface defects in catalytic processes relevant to fuel decomposition and combustion mechanisms, providing valuable guidance for future experimental and theoretical studies aimed at optimizing alumina-based catalysts and storage materials for energetic applications.

## Data Availability

No datasets were generated or analysed during the current study.
